# Learning Curve: Putting Healthy School Principles into Practice

**DOI:** 10.1289/ehp.117-a448

**Published:** 2009-10

**Authors:** Tina Adler

**Affiliations:** **Tina Adler** first wrote for *EHP* about the Clinton–Gore environmental agenda in 1993. She is a member of the National Association of Science Writers and the American Society of Journalists and Authors

The H-shape design planned for New York City’s new Public School 109, as described by *The New York Times*, allowed for large courtyards shielded from neighbors’ noise for play and recreation, windows that open onto the courtyards to provide light and air, and thoroughly ventilated wardrobes to dry clothing and maintain circulation. That was a few years ago—in 1901, to be exact. But those long-ago improvements—attention to indoor air quality, ventilation, lighting, and acoustics—now distinguish “high-performance” schools, which are specifically designed to promote better attendance, achievement, and behavior. Throw in energy and water conservation features—which are traditional “green” elements—along with a recycling program, an environmentally preferable purchasing program, nontoxic cleaning products, integrated pest management, a school garden to augment other healthful cafeteria food, and a sustainably developed site, and you have the new ideal for today’s healthy schools and child care centers.

But many children attend schools that bear no resemblance to this picture. Numerous studies have demonstrated that schools can be places where kids too often are exposed to toxic chemicals, mold, lead, asbestos, and other harmful agents. Moreover, some schools are located in areas where the outdoor air is so polluted that teachers wouldn’t want to open the windows even if they could. With children spending about one-third of their day at school, healthy school facilities could, if given the support, provide children with the most pollution-free part of their day, experts say.

## Making Sure Green Schools Are Healthy, Too

Today there is considerable overlap between green energy efficiency and resource conservation measures and the elements that characterize high-performance schools. For instance, according to the National Renewable Energy Laboratory, increased use of daylighting, which reduces use of artificial lighting, yields both energy savings and better test scores (a function, in part, of improved ability to concentrate). In the early years of the green school movement, however, agencies and architects focused almost entirely on energy efficiency, says Barbara Sattler, an environmental health professor at the University of Maryland School of Nursing. One of the leaders in the field, the U.S. Green Building Council (USGBC), had recently established its now well-known Leadership in Energy and Environmental Design (LEED®) for New Construction rating system, in which new buildings achieve one of various ratings depending on the number of energy efficiency features they incorporate. But when Sattler would speak up about making buildings healthy as well as energy efficient at early USGBC meetings, “people didn’t always understand what that might mean,” she says.

However, policy makers in Los Angeles—one city where the high-performance school movement first flowered and is now in full bloom—did understand. In the late 1990s, faced with severe overcrowding, the Los Angeles Unified School District sought and received funding for the largest new school construction program in the country. Ahead of its time, the LA Board of Education passed a resolution mandating that schools be built to standards outlined by the then–newly formed California-based Collaborative for High Performance Schools (CHPS). The CHPS criteria began with LEED for New Construction and added guidelines for additional elements that more directly related to student health as a means of fostering better performance. Where other green building groups focused primarily on energy and water use efficiencies, the Board’s stated priorities for adopting CHPS were to protect student and staff health and to maximize academic performance by providing a healthy environment.

The CHPS rating system certifies schools as being “high-performance” and awards points for energy efficiency, water conservation, natural lighting, thermal comfort, noise control, and ease of operation (to ensure the building is maintained well), among other features. CHPS offers two levels of certification. Schools can have their buildings rated and verified by an outside party, or they can “self-certify,” meaning they use a scorecard to ensure they meet CHPS design criteria.

Eleven states—including New York, Texas, Colorado, Washington, and the New England states—have adopted CHPS criteria as a voluntary program for their school districts. In addition, more than 40 districts now require that schools be designed using the CHPS criteria, with 30 of those signing on in the past two years, says Ariel Dekovic, communications manager for CHPS. Temporary or “relocatable” classrooms have their own set of CHPS guidelines for energy conservation, indoor air quality, lighting, and the design of their heating, ventilation, and air conditioning (HVAC) system. CHPS also offers a maintenance and operations manual for keeping high-performance features operating as intended.

In 2006 two frequently cited reports brought broader attention to the merits of combining “green” with “high-performance” in schools. *Greening American’s Schools: Costs and Benefits*, by Gregory Kats, and *Green Schools: Attributes for Health and Learning*, by the National Research Council, both described how the indoor environment affects children’s health and thus academic performance. Moreover, Kats calculated that “green schools” (which he defined to include some high-performance characteristics) cost on average about $3 more per ft^2^ to build than conventional schools. However, he calculated the direct and indirect health and productivity benefits of these same improvements—which included energy and water cost savings, greater teacher retention, and reductions in colds, flu, and asthma, among other benefits—to be about $74 per ft^2^.

In spring 2007 USGBC launched its LEED for Schools rating system for new construction and major renovation, adding an acoustic prerequisite, a mold prevention credit, and additional siting criteria to protect the health of both students and surrounding ecosystems, says Rachel Gutter, senior manager of USGBC’s education sector. For instance, schools can develop a master plan for their entire campus to conserve existing habitat, minimize the extent of impermeable surfaces (which exacerbate stormwater runoff), restore damaged areas, and use naturally pest- and drought-resistant native plants for landscaping. Schools can also earn a credit for making their playing fields and other facilities available for community use—a boon for communities with few public exercise options.

With the encouragement of USGBC, CHPS, and other groups and government agencies, many schools are switching to nontoxic cleaning products that produce less indoor air pollution. According to the U.S. Environmental Protection Agency (EPA), the volatile chemicals in many cleaners, floor waxes, disinfectants, and other janitorial supplies can irritate the eyes and respiratory tract and cause headache, even dizziness; mitigating these distracting symptoms helps both students and teachers perform better. Schools are also implementing integrated pest management practices, which replace the routine use of pesticides with specific cleaning and building maintenance measures. As of 2007 more than 70% of California school districts adopted integrated pest management practices.

In Washington, DC, schools are being renovated to LEED for Schools standards as part of a 15-year, $3.5-billion campaign to modernize all of the city’s 142 school buildings. The campaign is focused on creating a better environment for learning, says Christopher Dunlavey, program manager for the DC Office of Public Education Facilities Modernization and president of Brailsford & Dunlavey, a facility planning and program management firm. “We tried to home in exactly on what facility conditions are important to supporting academic performance,” he says.

But Marni Allen, senior research and policy associate for the 21st Century School Fund, which maintains a database of DC public school buildings, finds “a huge variation” in the condition of the DC schools. Some have been fully modernized, “and then you go look at the ones that haven’t been, and it can be pretty startling,” she says. She and her colleagues are worried the funds will not stretch to meet the needs of all the schools.

## Costs: Gray Cloud in a Green World?

Green schools don’t have to cost more, some advocates say. “By far the greatest barrier to building green schools . . . is the perception that they cost more,” says Gutter. In a 4 August 2009 article in the online trade publication *School Construction News* she wrote, “Throughout the country, integrated project teams are building green schools that don’t cost more and sometimes cost less [than conventionally designed facilities].”

“The construction cost of a green building does not have to be greater than a traditional one,” agrees Jane Rath, a principal with SMP Architects in Philadelphia and an expert in green design for schools. But some options, such as installing a vegetated “green roof” or one’s own power source, will cost more than the traditional alternative. Also, if LEED certification is pursued, costs can go up by $30,000–50,000, says Rath, who explains that building designers must charge more for the work to meet LEED certification requirements.

For his part, Dunlavey says incorporating LEED criteria used to add 3% to his design and construction costs, but that’s no longer the case. “Green design practices are becoming so common and expected . . . that those premiums are shrinking.” The premium depends to some degree on the individual builder’s experience with and sources for green building materials (for more information, see “Bringing Green Homes within Reach: Healthier Housing for More People,” *EHP* 116:A24–A31 [2008]).

Additional costs come through commissioning, the detailed testing and balancing of the school’s operation and maintenance systems. “A green building is only as good as its commission,” says Wayne Thomann, an assistant professor in the occupational and environmental medicine division at Duke University School of Medicine. And commissioning itself can cost another $50,000, according to Rath.

Despite the gains that Kats and others have calculated can accrue from such investments, getting districts to commit to building green schools can be challenging, because they generally want to build or renovate schools quickly and at as low a cost as possible, says Bob Axelrad, a senior policy advisor in the EPA Indoor Environments Division and chairman of the agency’s School Siting Workgroup. “The separation of capital budgets from operation and maintenance budgets in many districts means that future cost savings may not even be considered in school construction decisions,” he says.

One way to get around the problem: Schools without big budgets can implement small-scale high-performance and green features by selecting projects carefully and phasing them in systematically, says Claire Barnett, executive director of the nonprofit Healthy Schools Network, Inc. Simple improvements (some of which are already mandated by state law) include removing para-dichlorobenzene cake toilet deodorizers; installing “walk-off” mats at all major entries, which keeps schools cleaner and helps prevent pollutants being tracked indoors; ensuring air intakes are clear and open on the outside; prohibiting bus and car idling near the building; and phasing in the use of nontoxic cleaning products and pest control methods. Barnett notes that entities that are going to set guidelines for school design must ensure their advisory committees include advocates for children, teachers, custodians, and other occupants who have actual, not just theoretical, knowledge about how public schools actually operate day to day.

## Making Standards Work

Fundamental technical hurdles to building healthy schools still exist, particularly in the area of indoor air quality, researchers say. “We are making good progress . . . with striking that balance between saving energy and improving greening and indoor air quality and moisture management [to prevent mold]. But a lot of research has to be done,” says Thomann. He and his colleagues are revising a position paper for the American Society of Heating, Refrigeration and Air-Conditioning Engineers that addresses the challenge of saving energy and reducing greenhouse gas emissions while maintaining good indoor air quality, which he hopes to publish within a year.

A similar overarching question is whether the current rating systems address key environmental health concerns. Harvard University’s John Spengler, chairman of the committee that wrote *Green Schools: Attributes for Health and Learning,* asserts that because USGBC and CHPS must develop their criteria with input from many different interest groups that have conflicting concerns, they may be slower to take action on controversial materials such as flame retardants. It’s not that the groups can’t or won’t address these issues, he says, “but the way they are structured makes it a lot more difficult.”

Orr points out that improvements to the standards are ongoing. “CHPS and its stakeholders have pioneered approaches to daylighting, displacement ventilation, and low-emitting materials, and continue to pioneer the latest strategies in acoustics, indoor air quality, materials, and energy efficiency,” he says. “Sometimes these advances are not quite ready for incorporation, and they are deferred to the later updates of our standards.”

“To increase adoption of guidelines, these groups have to strike a balance between what is ideal and what is pragmatic,” notes Axelrad. The EPA is attempting to do just this by creating new guidelines on all aspects of school environmental health, from how to get rid of unnecessary toxic chemicals to managing mold and moisture and indoor air quality. The EPA created the comprehensive Healthy School Environments Assessment Tool (HealthySEAT) software program to help states and districts assess and manage their schools’ environmental and safety conditions. Many schools use HealthySEAT to develop indoor air management programs, says Axelrad. CHPS is also developing an operations report card for all schools to assess the major facets of operation.

Once measures are in place, however, realizing the benefits depends on proper building maintenance. “If you don’t have a trained workforce that is able to understand and manage and operate it, then you’ve really lost all the advantages of going through a rating system,” says Spengler.

When staff members from custodians to teachers aren’t properly trained, numerous problems can erupt. For instance, Thomann says, schools will shut down the HVAC system at night to save money but create another form of greening—mold growth. And in one North Carolina classroom, the teacher had filled the special “light shelves,” designed to bounce daylight further into the classroom, with her supplies, says Gutter. No one had told her what the shelves were for.

“We’ve seen so many times that the facility operators—the custodial services—can’t operate complex systems,” Spengler says. Building facilities have become unnecessarily complicated and high-tech, he and others say, and Barnett stresses that school facilities must be designed to be easy to maintain for indoor health. She also points out that schools facing steep budget cuts are reducing their facility and custodial staff.

Support for schools needs to come from the federal government, and no federal guidelines or legislation exist to fully support the healthy school movement, experts say. The principles of high-performance school design are spelled out in the No Child Left Behind Act of 2001, but Congress has not appropriated funds to help school districts implement these, says Barnett.

## The Budget Question

Members of Congress are considering school funding legislation that some advocates say is necessary for schools across the country to get healthier. In May 2009, the House of Representatives passed the 21st Century Green High-Performing Public Schools Facilities Act. The bill would allow the Department of Education to authorize billions of dollars for state and local educational agencies to conduct high-performance and green school projects. The Senate is still debating its version of the bill.

Advocates looked last winter to the federal stimulus package for support for high-performance schools. The American Recovery and Reinvestment Act was passed with $39.5 billion of its State Fiscal Stabilization Fund earmarked for uses including “modernization,” which could include green building renovations. Yet an August 2009 survey by the American Association of School Administrators revealed that only 12% of respondents were using the funds for modernization, whereas more than half were using the money to save personnel positions. “By next year,” Barnett says, “we will all know more about how [stimulus funds were] spent by local schools and states.”

Barnett notes there are also bond funds newly available as well as federal funds that go directly to local schools for use in renovations and repairs (but not new construction). Schools nationwide spend $20–30 billion per year on facility projects, she says, but still have decades’ worth of deferred repairs.

“We all know that budgets are zero-sum most of the time—if money has to go to [school improvements] it has to be taken from somewhere else, and the question is where?” says Sattler. As a society, she says, “we need to decide where our funding will go. It seems to me that taking care of our children by creating learning spaces that are healthy and safe would be a good reflection of our values.”

## When the Outside Air Comes In

A key school-related environmental health issue now on the EPA’s agenda is the siting of schools. “Without careful attention, building a great energy-efficient school near polluting facilities isn’t really doing anything for the health and safety of students,” says Renee Blanchard, campaign coordinator for the Virginia-based Center for Health, Environment and Justice (CHEJ), which is working to strengthen school siting policies. A 2002 CHEJ study documented in the report *Creating Safe Learning Zones* showed that more than 1,100 schools in New York, New Jersey, Massachusetts, California, and Michigan fell within a half-mile of a Superfund site. Moreover, in the September 2008 issue of the *Journal of Environmental Planning and Management*, Sergey Grinshpun and colleagues reported that about one-third of U.S. public schools are located within 400 m of a major roadway, and about one-tenth are within 100 m, exposing children to potentially hazardous traffic-related pollutants. The EPA is mandated to develop federal guidelines for the siting of schools, taking into consideration proximity to toxics and the availability of safe routes to schools. Draft school siting guidelines will be available for public comment in late 2009 or early 2010.

Meanwhile, on 31 March 2009, the EPA announced it would monitor the air quality outside 62 schools in 22 states as part of an initiative to better characterize risks posed to schoolchildren by air pollution. Since then, two tribal nation schools have been added to the list. Once the air toxics data for a school are confirmed, the EPA will estimate potential exposures and health effects. If a school is found to have low potential for long-term health effects, the agency may cease monitoring at that site. At sites where estimated health risks are high, the EPA will seek to mitigate the sources of pollution. The first monitoring data were posted in August 2009 at http://www.epa.gov/schoolair.

## For More Information

Collaborative for High Performance Schools, https://www.chps.net

U.S. Green Building Council, http://www.usgbc.org/

HealthySEAT, http://www.epa.gov/schools/healthyseat/

## Figures and Tables

**Figure:**
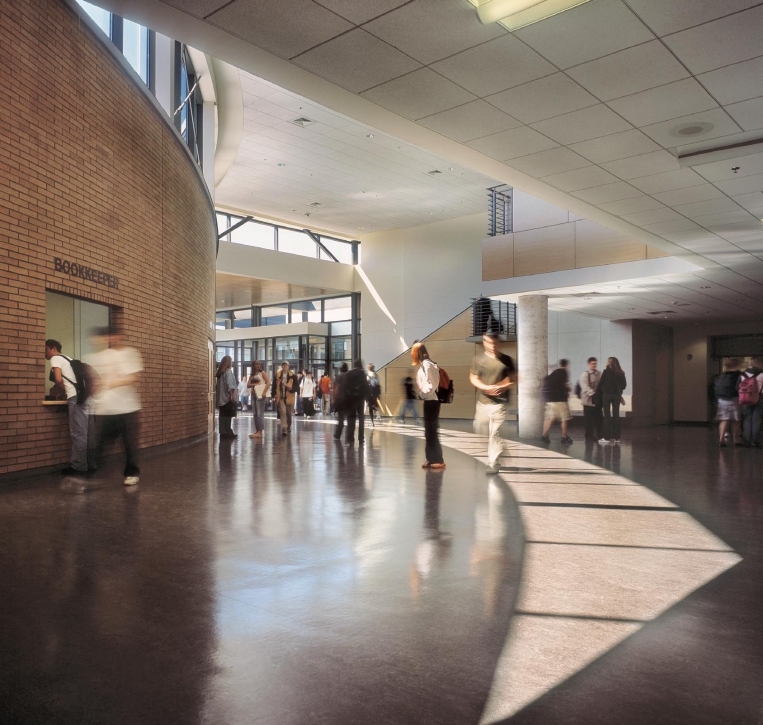
Clackamas High School, a public high school in Oregon, is considered a model healthy school.

**Figure:**
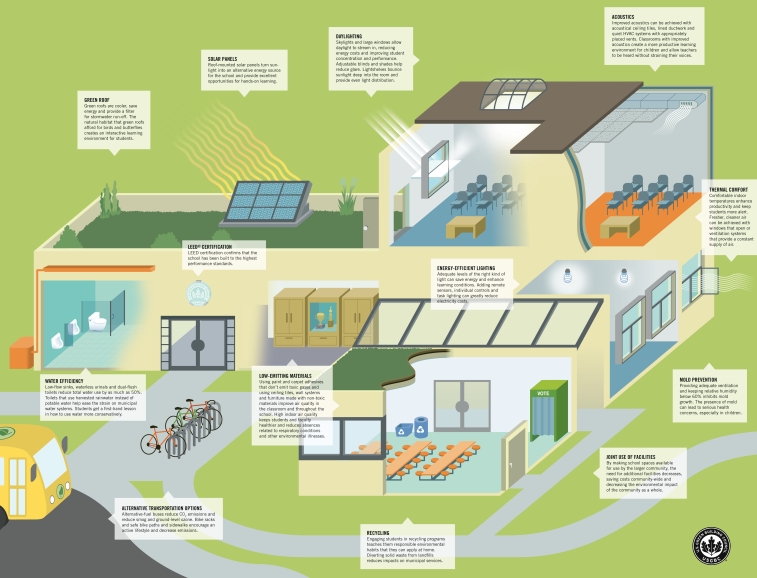
“Green” features, which typically focus on energy efficiency and resource conservation, are merging with “high-performance” elements designed to enhance student productivity to produce today’s ideal for a healthy school environment. Source: U.S. Green Building Council

