# Erratum

**Published:** 2009-12

In the October 2009 article “Learning Curve: Putting Healthy School Principles into Practice” [ Environ Health Perspect 117:A448–A453 (2009)], William Orr is quoted but never fully identified by name. Orr is executive director of the Collaborative for High Performance Schools. *EHP* regrets the omission.

